# Serotonin transporter promoter methylation in peripheral cells and neural responses to negative stimuli: A study of adolescent monozygotic twins

**DOI:** 10.1038/s41398-018-0195-6

**Published:** 2018-08-08

**Authors:** Elmira Ismaylova, Melissa L. Lévesque, Florence B. Pomares, Moshe Szyf, Zsofia Nemoda, Cherine Fahim, Frank Vitaro, Mara Brendgen, Ginette Dionne, Michel Boivin, Richard E. Tremblay, Linda Booij

**Affiliations:** 1 CHU Sainte-Justine Research Centre, Montreal, Canada; 2 Department of Psychiatry, University of Montreal, Montreal, Canada; 3 Department of Psychology, Concordia University, Montreal, Canada; 4 Department of Pharmacology and Therapeutics, McGill University, Montreal, Canada; 5 School of Psychoeducation, University of Montreal, Montreal, Canada; 6 Department of Psychology, University of Quebec à Montreal, Montreal, Canada; 7 School of Psychology, University of Laval, Quebec, Canada; 8 Institute of Genetic, Neurobiological, and Social Foundations of Child Development, Tomsk State University, Tomsk, Russian Federation; 9 Department of Psychology, University of Montreal, Montreal, Canada; 10 School of Public Health, Physiotherapy and Sports Science, University College Dublin, Dublin, Ireland

## Abstract

Several studies have examined associations between peripheral DNA methylation patterns of the serotonin transporter gene (*SLC6A4*) promoter and symptoms of depression and anxiety. The *SLC6A4* promoter methylation has also been associated with frontal-limbic brain responses to negative stimuli. However, it is unclear how much of this association is confounded by DNA sequence variations. We utilized a monozygotic-twin within-pair discordance design, to test whether DNA methylation at specific CpG sites in the *SLC6A4* promoter of peripheral cells is associated with greater frontal-limbic brain responses to negative stimuli (sadness and fear), independently of DNA sequence effects. In total 48 pairs of healthy 15-year-old monozygotic twins from the Quebec Newborn Twin Study, followed regularly since birth, underwent functional magnetic resonance imaging while conducting an emotion-processing task. The *SLC6A4* promoter methylation level was assessed in saliva samples using pyrosequencing. Relative to the co-twins with lower *SLC6A4* promoter methylation levels, twins with higher peripheral *SLC6A4* methylation levels showed greater orbitofrontal cortical (OFC) activity and left amygdala-anterior cingulate cortex (ACC) and left amygdala-right OFC connectivity in response to sadness as well as greater ACC-left amygdala and ACC-left insula connectivity in response to fearful stimuli. By utilising a monozygotic-twin design, we provided evidence that associations between peripheral *SLC6A4* promoter methylation and frontal-limbic brain responses to negative stimuli are, in part, independent of DNA sequence variations. Although causality cannot be determined here, *SLC6A4* promoter methylation may be one of the mechanisms underlying how environmental factors influence the serotonin system, potentially affecting emotional processing through frontal-limbic areas.

## Introduction

Animal and human studies suggest that environmental exposures can affect our epigenome and these epigenetic marks can persist through cell divisions^1^. Epigenetic modifications can change gene expression without altering the genetic sequence. These epigenetic modifications play an important role in setting up tissue- and cell type-specific gene expression programs during development and are crucial in maintaining normal cellular physiology^2^. DNA methylation—as one of these possible epigenetic mechanisms—implies a covalent modification of the DNA molecule itself through enzymatic addition of a methyl group to (mostly) cytosine bases^2^. DNA methylation, involved in epigenetic regulation of gene expression, at critical regulatory regions such as promoters and enhancers, can lead to permanent silencing of the gene, either directly by blocking transcriptional factors from binding to the DNA sequence or by attracting proteins to form corepressor complexes^3,4^.

DNA methylation patterns are shaped during development and cellular differentiation but are also responsive to environmental signals, particularly during gestation^5^. Indeed, even in newborns, variations in DNA methylation levels can be detected across twins, suggesting that environmental factors might be influencing the normal developmental trajectories of DNA methylation pattern^6^. DNA methylation alterations in response to early environmental exposure also appear to be stable, at least in some gene regions, for many years. For example, a study in humans has shown that individuals who were exposed to the Dutch famine in the perinatal period had, six decades later, altered DNA methylation in particular sites compared to their non-exposed siblings^7^.

One of the most widely studied genes in relation to DNA methylation and mental health is the serotonin transporter gene, *SLC6A4*. The serotonin transporter is of interest as it has been shown to play an important role in mood, cognition and psychophysiology, as well as brain development^8^. Following results of association studies showing a link between *SLC6A4* methylation assessed from peripheral cells and early life environment and later behavior in human clinical and nonclinical populations^9,10^, a number of studies have now investigated possible associations between peripheral *SLC6A4* methylation and brain processes. For instance, Wang et al.^11^ found that *SLC6A4* promoter methylation in T lymphocytes of adults was associated with lower in vivo Positron Emission Tomography (PET) measures of brain serotonin (5-hydroxytryptamine; 5-HT) synthesis in the orbitofrontal cortex and higher childhood aggression. Furthermore, a recent study found associations between *SLC6A4* methylation and in vivo PET measures of serotonin transporter availability^12^. We also previously reported that *SCL6A4* methylation obtained from white blood cells was significantly associated with activation in response to negative emotional stimuli in the insula^13^ and lower hippocampal volume^14^ in depressed patients and controls. Using functional magnetic resonance imaging (fMRI), greater peripheral *SLC6A4* methylation from saliva and whole-blood DNA samples has also been associated with an increased amygdala response to threat-related stimuli in adolescents and young adults^15,16^. In one (f)MRI study, we reported that greater peripheral *SLC6A4* methylation from whole-blood, saliva, and buccal DNA samples have been associated with greater (superior) prefrontal cortical GM volume and parietal-frontal regional functional connectivity at rest in healthy adults^17^. Together, these findings support the relevance of peripheral *SLC6A4* methylation measures for frontal-limbic brain processes and support the notion that *SLC6A4* methylation level changes may be an underlying mechanism of how environmental factors can influence aspects of the 5-HT system and, consequently, emotion processing.

However, these associations could be confounded by the effects of variations in DNA sequence. Indeed, certain genotypic variants have been shown to have different DNA methylation patterns than others in the context of similar exposures and experiences^18^. For instance, lower methylation was found in association with unresolved trauma or loss in individuals with the s/s genotype of the *SLC6A4* promoter polymorphism, while an inverse correlation was found in the l/l genotype group^18^. Furthermore, the influence of DNA sequence on methylation status may depend on genomic location, cell type, and developmental stage^19^.

Monozygotic (MZ) twin study designs are unique in their ability to control for DNA sequence differences because each pair of MZ twins shares essentially the same genetic sequence. Thus, differences within twin pairs in gene expression and phenotype, including brain processes, can be attributed to environmental effects rather than DNA sequence influences.

In the present study, we utilized a MZ design to test, in a sample of healthy adolescents, whether DNA methylation at the *SLC6A4* promoter in peripheral cells is associated with greater frontal-limbic activation and connectivity in response to negative stimuli, irrespective of DNA sequence differences. Specifically, we focused on sad and fearful stimuli because neural responses to these negative emotional facial expressions have been relatively consistently associated with *SLC6A4* genetic and epigenetic variation^13,15,20^.

## Subjects and methods

Participants were 96 monozygotic twins (48 pairs: 21 male and 27 female pairs; age 15 years) from the Quebec Newborn Twin Study (QNTS)^21,22^, followed longitudinally since birth, in the area of Montreal, Canada, between April 1995 and December 1998. The number of individuals (*n* = 96, 48 pairs) was larger than the sample size of our previous studies in non-twins and that focused on *SLC6A4* methylation and its link with brain processes (e.g., 13, 17). They were all physically healthy, free of any medication liable to affect brain function and free of current and past psychopathology. Mental health status was confirmed by the Schedule for Affective Disorders and Schizophrenia for School-Age Children- Present and Lifetime Version (K-SADS)^23^. Furthermore, we used the Dominic Interactive (Adolescent version), a computerized questionnaire to assess subclinical levels of internalizing symptoms (phobias, generalized anxiety and depression)^24^. The appropriate ethics committees (CHU Sainte-Justine and Montreal Neurological Institute) approved the research protocol. Written assent was obtained from all participants and written consent from parents of all participants.

### DNA methylation protocol

Whole saliva was collected using the Oragene TM DNA self-collection kit following manufacturer instructions (DNA Genotek Inc., 2004, 2006). Participants did not eat, chew gum, or drink anything but water for 30 min prior to saliva collection. Once extracted, whole-saliva DNA was converted with EZ DNA Methylation-Gold Kit (D5006, Zymo Research, Irvine, CA, USA). Following recommendation by Tost and Gut^25^, 20 ng of bisulfite-treated DNA was used in the PCR amplification to reach high reproducibility of pyrosequencing, using previously published primer sets optimized for bisulfite-treated DNA template (11, Fw: 5′TTGTTAGGTTTTAGGAAGAAAGAGAGA-3′, Rev: 5′Biosg-AAAAAAACTACACAAAAAAACAAATATAC-3′) with EpiMark Hot Start Taq DNA Polymerase (New England Biolabs, Ipswich, MA, USA). Ten CpG sites (numbered as 5–14) in the 28563060–28563221 region of chromosome 17 (according to hg19) were assayed.

Site-specific methylation analyses were performed by pyrosequencing using PyroMark Q96 (Qiagen, Venlo, Limburg, Netherlands) using the CFI Imaging and Molecular Biology Platform at McGill University in the Department of Pharmacology and Therapeutics using 2 sequencing primers: 5′AAGAGAGAGTAGTTT-3′ and 5′GTAGATTTTTGTGTG-3′. The DNA methylation level at each CpG site was computed after quality control of the triplicates. If a sample did not pass quality control at a certain CpG site, the mean DNA methylation value for that CpG site was calculated from the remaining two replicates. We then computed the average methylation level from our ten CpG sites of interest (CpG sites 5–14). In line with other studies that linked brain processes with *SLC6A4* methylation^13,16,17,26^, average methylation levels across the investigated CpG sites values were calculated and used in the analyses. The average value of promoter methylation level was used in the analyses rather than the DNA methylation level of each individual CpG site to limit the number of statistical comparisons. The choice to calculate the mean of the ten studied CpG sites was further based on our previous work in healthy and clinical samples, showing associations between average *SLC6A4* methylation levels across these 10 sites and in vivo measures of brain 5-HT synthesis in the frontal-limbic brain circuits as well as frontal-limbic functional and structural processes^11,13,17^.

### Neuroimaging

Participants were scanned on a 3 T Siemens TIM Trio Scanner located at the Montreal Neurological Institute (MNI) with a 32-channel head coil. The scan included a magnetization-prepared rapid acquisition gradient-echo (MPRAGE) 9 min sequence (176 slices, 1 mm thickness, TR = 2300 ms, TE = 2.98 ms, TI = 900 ms, FA = 9°, FOV = 256 × 256 mm, matrix size 256 × 256) to assess brain anatomy. Next, a 15-min functional scan during an event-related emotion-processing task, adapted from the task used in Canli and colleagues^27^, was performed. Briefly, the task consisted of a series of 120 Ekman faces with different emotions (happy, sad, angry, fearful, and neutral) from the Pictures of Facial Affect series^28^. Faces were presented randomly for 2 s and followed by a fixation cross (~2 s; jittered) and a question asking whether the face belonged to a man or a woman. Two hundred and 26 functional whole-brain images (multi-slice gradient echo EPI with 3.5 mm isotropic resolution and TR/TE = 2110/30 ms, 40 slices, FOV = 224 × 224 mm, matrix size 64 × 64, interleaved descending slice acquisition) were acquired.

### Image processing

#### Task-based fMRI

Pre-processing steps (slice timing and motion correction, coregistration with the anatomical scan, normalization into an EPI stereotactic Space (MNI template) and spatial smoothing at 7 mm FWHM with a Gaussian Kernel were performed in SPM8 (Wellcome Department of Cognitive Neurology, London UK). For each emotion, activation during neutral stimuli was subtracted from the activation during the different emotions. Voxel-wise sad minus neutral and fear minus neutral contrasts were generated at the first level. We calculated within-pair discordance by subtracting the activation contrast of the twin with higher *SLC6A4* methylation levels from the activation contrast of the co-twin with lower *SLC6A4* methylation levels^29^. Discordance values were then used in the regression analyses, by regressing discordance in brain activation in response to sadness>neutral and fear>neutral independently onto discordance in methylation. Whole-brain analysis was conducted. The threshold *p* value was set at *p* < 0.001. Only clusters showing a spatial extent of at least 10 contiguous voxels were considered (except for the amygdala, in which we required at least 5 contiguous voxels). We corrected for multiple comparisons at the cluster level using the family-wise error rate (FWE)^30^.

#### Functional connectivity

Following the pre-processing steps, functional connectivity analyses were performed using the CONN Functional Connectivity toolbox v174 (http://www.nitrc.org/projects/conn). We controlled for the possible confounding effects of head motion artifacts using the ComCor strategy and default preprocessing parameters^31,32^. In addition, we applied the following denoising options in CONN: detrending, which removes linear/quadratic/cubic trends within each functional session, and despiking, which applies a squashing function to reduce the influence of potential outlier scans^32^.

We conducted ROI-to-ROI analyses to assess functional connectivity during sad and fearful conditions. The following ROIs were chosen, namely bilateral amygdala, bilateral orbitofrontal cortex (OFC), anterior cingulate cortex (ACC) and bilateral insula. Amygdala was chosen in light of the link between peripheral *SLC6A4* methylation and amygdala responses to fearful stimuli in (non-twin) adolescents^16^. OFC was of particular interest in light of the results of our study in healthy (non-twin) individuals showed an association between lower brain serotonin synthesis in the OFC and greater peripheral *SLC6A4* methylation^11^. ACC was chosen given the findings of activation in this region during sadness in (non-twin) individuals with depressive symptoms^33,34^. Insula was chosen based on our previous study showing an association between insula response to negative stimuli and peripheral *SLC6A4* methylation in (non-twin) healthy and depressed adults^13^. Mean BOLD time series were extracted from each ROI and correlated with the BOLD time-series signal of every other ROI in the network to create a ROI-to-ROI connectivity matrix (connectome) showing connectivity between each region within the network.

In order to assess discordance between ROI-to-ROI connectivity values based on peripheral *SLC6A4* promoter methylation discordance, ROI-to-ROI connectivity for twins with greater *SLC6A4* methylation level was compared to their co-twins with lower methylation level. Given the sample size and the correlational nature of functional connectivity and to balance the risk for type I and type II errors, we set the statistical threshold for each voxel at *p* < 0.05, uncorrected.

## Results

### DNA methylation

The mean *SLC6A4* promoter methylation level was 3.74% (SD = 2.37, range: 1.23–14.37%). This level is comparable to that found in other peripheral *SLC6A4* promoter methylation studies, derived from various biological tissues including saliva, using the same CpG sites^11,13,17^. Within pair, the average discordance in *SLC6A4* promoter methylation varied from 0.23 to 8.58%. There were no sex differences in mean *SLC6A4* methylation levels (F(1,94) = 0.01, *p* = 0.98), nor in *SLC6A4* methylation discordance levels (F(1,46) = 2.53, *p* = 0.12). Mean level of internalizing symptoms on the Dominic scale was 10.30 (SD = 6.16, range 1–27 out of maximum total score of 52), indicative of absent/low likelihood of internalizing symptomatology. Within pair, the average discordance in symptoms was 4.37 points (SD = 3.39, range 0–15). *SLC6A4* promoter methylation did not correlate with internalizing symptoms (as assessed by the Dominic Interactive questionnaire) neither globally (*r* = 0.12, *p* = 0.25) nor within pair (*r* = 0.12, *p* = 0.42).

### fMRI results

#### Sad condition

**Fig. 1:**
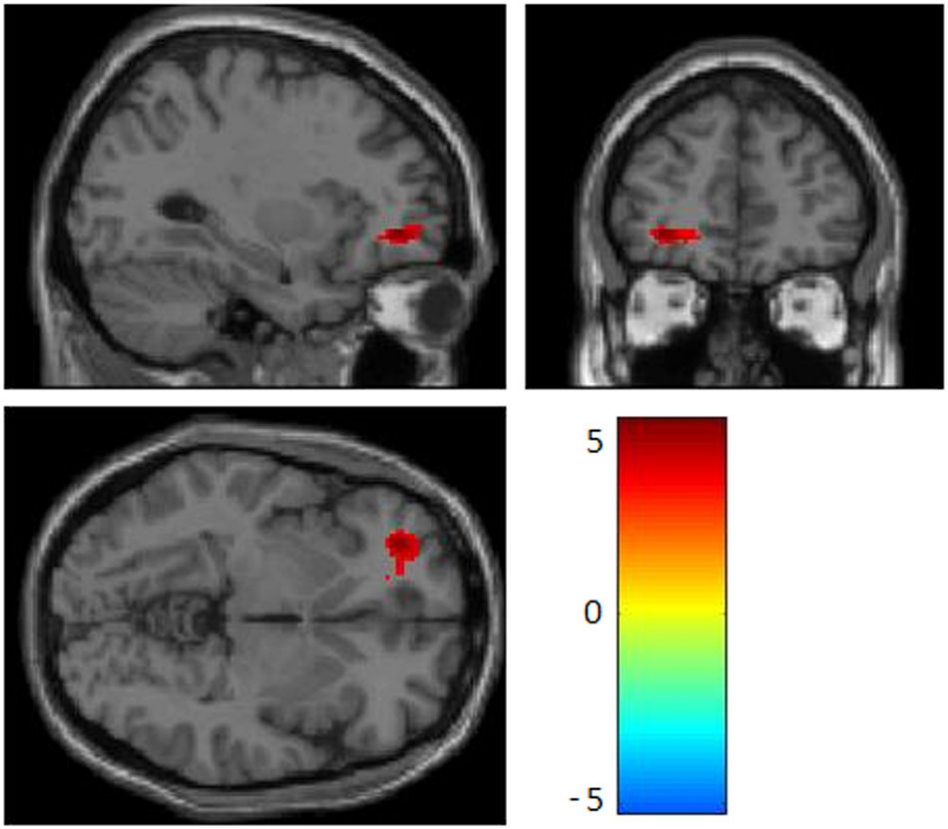
Greater within-pair difference in peripheral *SLC6A4* promoter methylation was associated with greater responses to sad stimuli in left orbitofrontal cortex (T = 5.11, pFWE = 0.032). The color bar represents T values

**Fig. 2:**
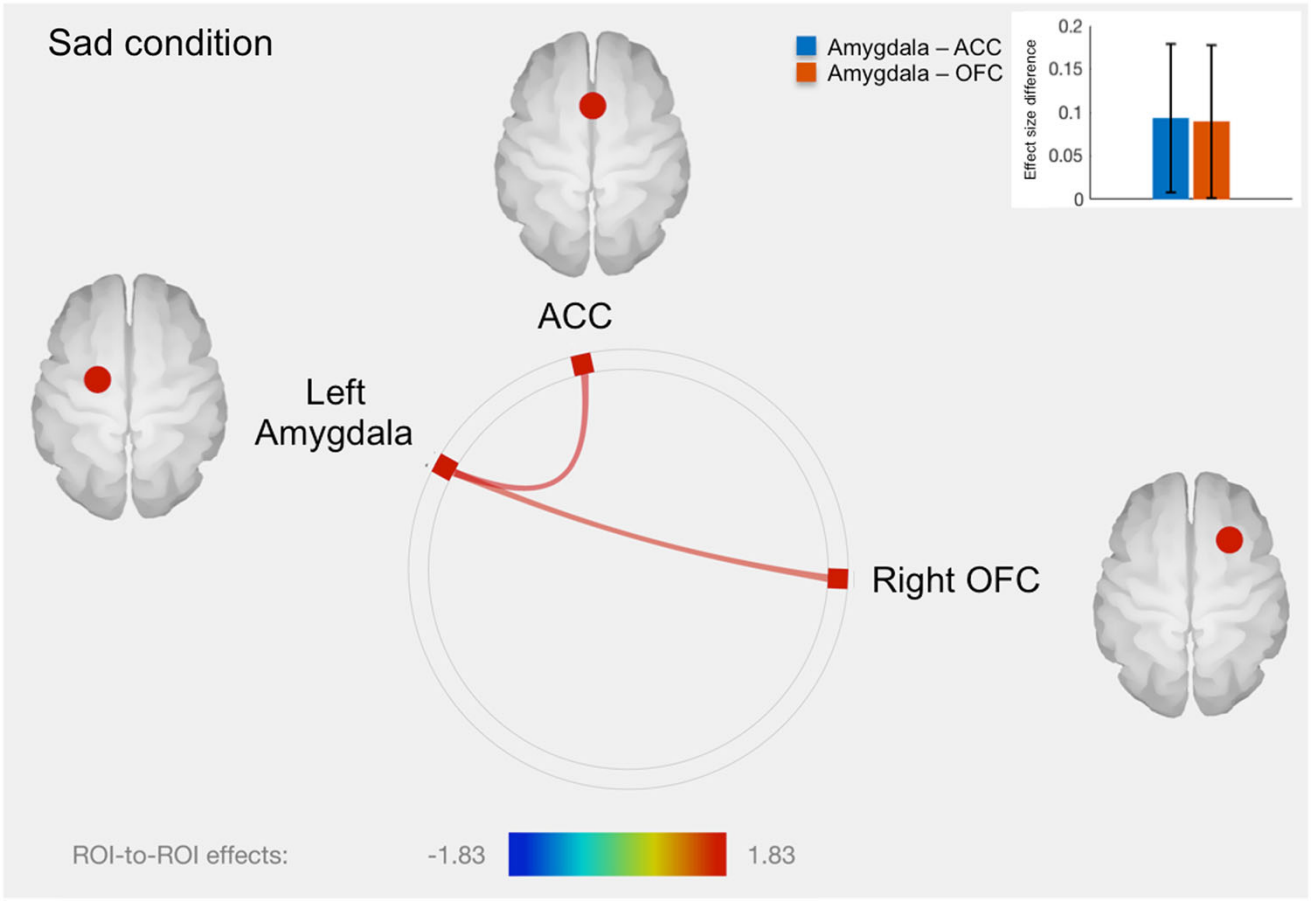
Connectome ring representation of ROI-to-ROI connectivity between amygdala, anterior cingulate cortex (ACC), orbitofrontal cortex (OFC) and insula in the sad condition. Only significant connectivity discordances are represented: greater ROI-to-ROI connectivity in the sad condition for twins with greater methylation level compared to their co-twins with lower methylation level, between left amygdala and ACC (T = 1.83, *p* = 0.036), as well as between left amygdala and right OFC (T = 1.71, *p* = 0.047). The color bar represents T values. The bars plot in the top right-hand corner represents differences in effect sizes (difference in Fisher-transformed correlation coefficients) between twins with greater methylation level and their co-twins with lower methylation level, for each connection

Relative to their co-twin with lower methylation, twins with higher *SLC6A4* promoter methylation levels had greater left OFC activation (*k* = 206, *t* = 5.11, cluster *p*_FWE_ = 0.03) (Fig. 1), as well as greater functional connectivity between left amygdala and ACC and between left amygdala and right OFC (Fig. 2 and Table 1). No other significant fMRI results were found in the sad condition.

**Table 1:** Significant within-pair fMRI associations with *SLC6A4* promoter methylation

Direction of effect: Condition	Region	*t*	*p*-value
Positive: Sad	left amygdala-ACC	1.83	0.036
	left amygdala-right OFC	1.71	0.047
Positive: Fearful	ACC-left amygdala	2.79	0.004
	ACC-left insula	1.85	0.035

*fMRI* functional Magnetic Resonance Imaging, *SLC6A4* serotonin transporter gene, *OFC* orbitofrontal cortex, *ACC* anterior cingulate cortex

#### Fearful condition

**Fig. 3:**
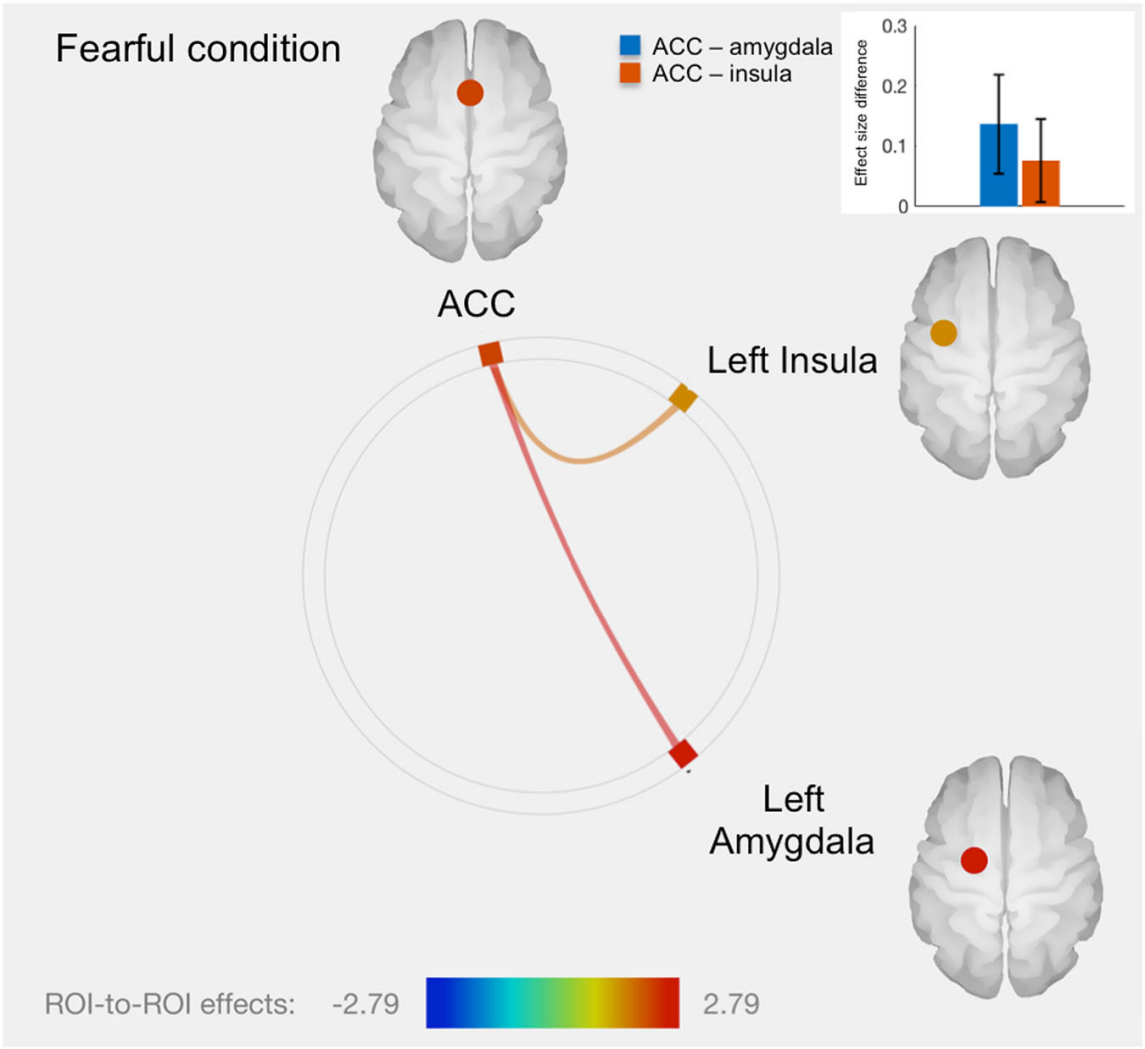
Connectome ring representation of ROI-to-ROI connectivity between anterior cingulate cortex (ACC), left amygdala and insula in the fearful condition. Only significant connectivity discordances are represented: greater ROI-to-ROI connectivity in the fearful condition for twins with greater methylation level: between ACC and left amygdala (*T* = 2.79, *p* = 0.004), as well as between ACC and left insula (*T* = 1.85, *p* = 0.035). The color bar represents *T* values. The bars plot in the top right-hand corner represents differences in effect sizes (difference in Fisher-transformed correlation coefficients) between twins with greater methylation level and their co-twins with lower methylation level, for each connection

There was no significant association with neural activity in the fearful condition. However, twins with higher *SLC6A4* promoter methylation level had greater functional connectivity between ACC and left amygdala and between ACC and left insula, relative to the co-twin with lower SLC6A4 methylation (Fig. 3 and Table 1). No other significant fMRI results were found in the fearful condition.

## Discussion

The aim of the present study was to assess potential neural correlates of peripheral *SLC6A4* promoter methylation during processing of negative facial expressions, i.e., sadness and fear. While an emerging number of studies have now shown that peripheral *SLC6A4* methylation correlates with frontal-limbic processing of negative emotions, it is not clear whether this association is due to the environment or to genetic factors. Here, we utilized a within-pair MZ-twin design to control for the putative role of DNA sequence in this association. We found that relative to the co-twins with lower *SLC6A4* promoter methylation, twins with greater *SLC6A4* methylation levels had greater left OFC activation, greater amygdala-OFC and left amygdala-ACC connectivity in the sad condition, as well as greater ACC-amygdala and ACC-insula connectivity in the fearful condition. In spite of relatively low within-pair variability, current findings are of particular interest given the implication of these brain regions in emotion regulation. Indeed, regional functioning of OFC, ACC, insula and amygdala, all part of the frontal-limbic circuitry, has previously been associated with emotion regulation^35,36^ and *SLC6A4* methylation^16^. Our findings do not only support the previously suggested hypothesis that peripheral *SLC6A4* promoter methylation shares potentially functional relevant information about frontal-limbic functioning and emotion-processing, but, most importantly, demonstrate for the first time that these associations occur, at least in part, independently of DNA sequence.

The finding of within-pair differences in neural processing of sad stimuli in the OFC regions fits well with the results of our previous study in which we compared neural activation to sad stimuli between 8-year-old monozygotic (MZ) and dizygotic (DZ) twins, allowing separation of genetic factors from environmental factors shared between twin pairs and non-shared environmental factors that are unique for each twin member^37^. Among other brain areas, neural activation during processing of sad stimuli in Broadmann Area (BA) 47, a key region in the OFC, was found to be fully driven by non-shared/unique environmental factors^37^. Interestingly, results of a positron emission tomography (PET) study, investigating the neural basis of various emotions (sadness, happiness, anger and fear) in healthy individuals, showed that the orbitofrontal regional activity was found to peak solely during the sadness condition^38^, emphasizing the role of the OFC in the detecting and processing of sad stimuli. That being said, in addition to the OFC, Damasio and colleagues^38^ reported increases in insula and ACC activity during sad condition (and, respectively, during fearful and angry condition). The absence of these insula-ACC regional responses to sad or fearful cues in the current study may be, in part, explained by differences in methodology (e.g., use of current implicit emotion-processing task versus self-generated emotional conditions by recall).

Furthermore, we previously found—using in vivo PET measures of brain 5-HT synthesis—that serotonergic synthesis in the OFC is not only modulated by DNA sequence^39^ but also by early environmental factors^40^. The environmental influences on serotonin functioning in the OFC observed in the present study also fits well with our previous PET study showing that greater peripheral *SLC6A4* methylation correlated with lower in vivo brain serotonin synthesis in the OFC region^11^. Current findings are also in line with the results of a study by another research group reporting positive association between peripheral *SLC6A4* promoter methylation and resting-state functional connectivity between amygdala, insula and ACC in healthy adults^26^. Notably, OFC, ACC, insula and amygdala constitute an integral part of the salience functional network, involved in affective processes^41^. Interestingly, results of an fMRI study examining neural correlates of emotion regulation in healthy individuals, showed that functional connectivity between amygdala and several (pre)frontal regions, including OFC and ACC, was associated with attenuated emotional response to negative emotional stimuli^41^, thereby underscoring the involvement of frontal-limbic functional connectivity in processing and regulation of emotions. Interestingly, salience network has been found to show relatively low cross-twin correlation in both MZ and DZ twins^42^, indicative of scarce evidence for either genetic or non-shared environmental effect on this network. Results of the present study suggest that this *SLC6A4*-related salience network is, at least in part, independent of variation in DNA sequence. Altogether the findings suggest an important role for OFC, ACC, and amygdala in the processing of sadness and fear, which appear to be partly driven by *SLC6A4*-modulated non-shared/unique environmental factors.

Some laterality effects were observed as well. Notably, the finding that the association in the amygdala was specific for the left side is in line with other research showing that the left amygdala is more frequently activated in response to emotional stimuli than the right amygdala (see ref. ^43^ for meta-analysis). Furthermore, stronger functional connectivity between left (but not right) amygdala and bilateral ACC and OFC has been associated with decreased negative affect induced by negative emotional content in healthy individuals^44^. Besides, conjoint activity of ACC and left (but not right) insula has been linked to increased awareness of one’s own emotions^45^. Interestingly, greater *SLC6A4* promoter methylation has been previously linked to greater left (but not right) amygdala and insula activation in response to negative emotional stimuli^13,16^. In light of these findings, we might advance that these lateralized regional activities reflect serotonin functioning-related neurobiological processing of the (sad or fearful) emotionally salient content. However, additional *SLC6A4* methylation fMRI studies of the connectivity of amygdala, OFC and insula subdivisions are necessary to deepen the understanding of the laterality.

While we found significant associations between peripheral *SLC6A4* promoter methylation discordance and neural processing of negative stimuli, we did not find any correlations between depressive symptoms and peripheral *SLC6A4* methylation. However, the levels of symptomatology variation were very low. The observation of an association between *SLC6A4* methylation and neural processing of negative stimuli in the absence of an association with anxiety- or depression-related symptoms is consistent with studies that include healthy individuals (e.g., 16). Thus, variation in frontal-limbic brain activation, as a function of variation in *SLC6A4*, occurs in the absence of behavioral variation. This supports the idea that *SLC6*A4 methylation could reflect a marker for neural regulation of negative stimuli, but additional factors may be necessary for an overt behavioral phenotype to be expressed^8^. Nevertheless, it would be of particular interest to test MZ twins who are highly discordant in levels of internalizing symptoms and in *SLC6A4* promoter methylation and link their discordance to differences in frontal-limbic processes.

Furthermore, peripheral *SLC6A4* promoter methylation levels and methylation differences within MZ twin pairs were low. However, considering the binary (either methylated or unmethylated allele at a particular CpG site) nature of the DNA methylation, the percentage change in DNA methylation indicates the fraction of cells having possibly experienced a complete change in methylation at a given site^17^. In other words, a small increase in *SLC6A4* promoter methylation of a tissue sample may suggest that few cells are potentially turning off their expression. Indeed, using an in vitro model, higher DNA methylation levels in the selected *SLC6A4* promoter region have been previously shown to lower its transcriptional activity^11^, thereby corroborating the regulatory role of DNA methylation of this region. In addition, peripheral DNA methylation in this particular gene region has been shown to be a potential marker of serotonin synthesis in the OFC^11^.

The strengths of this study include the use of a well-characterized, prospective, homogeneous longitudinal sample of adolescents. All twins were 15 years of age and carefully screened for the absence of psychiatric history. Furthermore, the investigated *SLC6A4* region and primary CpG sites were chosen a-priori, based on previous work and validation in vivo and in vitro in three independent samples^11,13,14,17^. However, the present findings should also be considered in the context of certain limitations. First, methylation was assessed from peripheral tissue (i.e., saliva), as DNA methylation generally cannot be assessed in the living human brain. However, studies combining epignegative stimuli are detectable while controlling for DNA sequenceenetics and various fMRI measures have shown substantial correlations between methylation assessed from peripheral tissue and brain functioning^13–17,46^. Moreover, future studies will be necessary to investigate potential moderating roles of age, sex, and various psychosocial variables, as well as to identify individual participant characteristics that account for the within-pair variation. Furthermore, we will continue to follow these twins in the future to assess the clinical relevance of these alterations in peripheral *SLC6A4* promoter methylation and processing of negative emotional stimuli. In spite of these limitations, the findings in our MZ twin study provide evidence that previously reported associations between peripheral *SLC6A4* promoter methylation and frontal-limbic processing of negative stimuli are detectable while controlling for DNA sequence. These associations may have implications for the future use of non-invasive DNA methylation markers in diagnosis, treatment and risk prediction in mental health problems in which serotonin plays an important role.
